# Medical Opinion and Movement

**Published:** 1910-01-15

**Authors:** 


### Medical Opinion and Movement.

At a meeting of the Société Médicale des Hôpitaux, Dr. Achard gave the results of some interesting observations on the effect of Anæsthetics upon the resistance and activity of the white corpuscles. *In vitro* cocaine and stovaine, even in very weak solutions of 1 in 1,000 and 1 in 2,000, diminish their activity. The activity of the white corpuscles is also much diminished by the vapours of bromide of ethyl, chloroform and ether. *In vivo*, in conditions of spinal anæsthesia, the leucocytes are hardly affected by stovaine, but their activity is weakened by cocaine in large doses. Ethyl chloride, the action of which is only of short duration, produces no change. Anæsthesia with ether and with chloroform lowers the resistance and activity of the leucocytes markedly for several hours. The corresponding properties of the serum are not always diminished in the same way, and the author therefore supposes that the anæsthetic fixes itself upon the white corpuscles. He suggests that surgeons might take these facts into consideration in the interpretation of certain accidents following operations under anæsthesia.

Drs. F. Berger and J. Tsuchiya have been conducting research on the Pathology of Pernicious Anæmia. They find that in cases of this disease the mucous membrane of the stomach and intestines contains a lipoid substance which can be extracted with ether, and possesses hæmolytic properties ten times as great as a similar lipoid from normal mucous membrane. Administered to animals, this lipoid has a small but definite anæmia-producing power, whether ingested or injected, whereas the action of the lipoid from a normal mucous membrane is much less marked. The anæmia thus obtained experimentally is of the pernicious type. After artificially producing a gastric or intestinal catarrh in dogs, the authors have obtained a similar lipoid with the same hæmolytic properties as in the cases of pernicious anæmia. The authors draw from their experiments the conclusion that the so-called "cryptogenic" forms of pernicious anæmia are due to the hæmolytic action of certain lipoids, accompanied by secondary insufficiency of the bone marrow. These lipoids appear to be formed in the gastro-intestinal mucous membrane, and are probably called into existence by a chronic catarrh, in which the different parts of the alimentary tract may more or less participate.

Dr. Paul Carnot contributes an article to *Le Progrès Médical* on the Therapeutic Use of Insufflations of Gas into the Stomach and Intestines. Gases may be introduced into the stomach by means of a tube communicating with a gas reservoir or bag. The pressure with which the gas enters should be measured by a manometer and should not exceed 20 centimetres of water. The gas gradually distends the stomach, and should be stopped as soon as the patient experiences a feeling of discomfort from gastric tension. The stomach remains distended for a certain time, and then the pylorus relaxes spasmodically and the gas enters the duodenum. The passage of the gas through the intestines is indicated by a series of gurglings, and some hours afterwards there is an abundant evacuaton of gas. Rectal insufflations may be carried out in a similar manner. The nature of the gas used depends upon the effects it is desired to produce. Oxygen has been chiefly employed; it increases the "respiration" of the gastro-intestinal cells and stimulates their activity, and at the same time it is inhibitive to the anærobic microbes of the intestines. Carbonic acid gas has a stimulating effect, and nitrogen may be employed in cases where a mechanical effect is desired without any chemical excitation. The indications for this method of treatment are briefly: Conditions of gastralgia, in which the insufflation causes a separation of the hypersensitive and irritated gastric mucous membranes by forming a buffer of gas between them. Cases of atony of the stomach and intestine, in which gaseous insufflations excite the musculature and increase the peristalsis. In such cases rhythmic insufflations are especially indicated. The oxygen bag is only half filled and by rhythmic pressure the gas can be made to flow backwards and forwards into the stomach, and in this way a sort of gastric gymnastic may be carried out.

Pathological research has placed practically beyond dispute the fact that Bacterial Infection is the prime causal factor of Thrombosis and Embolism following surgical operations. But the chief question that remains to decide is the ways and means by which this particular form of infection takes place. Professor J. Veit, of Halle, has recently published some observations on the subject, and he points out that even when every aseptic precaution is taken, if in the course of operation some cavity is opened, which may not be free from infective germs, and there are gaping vessels in the vicinity, they may become inoculated, and the accidents of thrombosis and embolism follow. In a series of 4,000 major gynæcological operations performed at Halle between 1900 and 1908 there were 25 cases of fatal pulmonary embolism. Among these there were only three hysterectomies for fibroma and three ovariotomies. Four of these operations were followed by a rise of temperature suggesting an accidental infection, but in all the remaining 21 cases there were no such indications. They all, however, involved the opening of a cavity with microbic possibilities, usually the vagina. In eight cases of hysterectomy for cancer, in which the details of the operation are fully reported, the author finds that hæmostasis was not complete before the vagina was incised, and he proceeds to show how the application of forceps and ligatures and such like manœuvres to open vessels with an adjoining infecting mucous membrane may easily lead to venous inoculation and thrombosis. In the operation of hysterectomy, therefore, Dr. Veit holds that one of the most important principles of procedure consists in securing complete hæmostasis of all the surrounding parts, and reserving the incision into the vagina as the last step in the operation, and similar rules should be laid down for other like operations, such as appendectomy, in which the appendix should only be incised or removed after the meso-appendix has been securely ligatured.

---

RAT-BITE Fever is the name suggested by Dr. T. J. Horder for a very curious syndrome of which he is able fully to report three cases in the *Quarterly Journal of Medicine*. This he believes to be a specific disease hitherto undescribed, at least in England, and the points in common to the three patients upon which this opinion is based are as follows. A bite from a rat is sustained, which may or may not suppurate at the time, though in one case it is noted that a similar bite from a ferret sustained the same day healed up much more quickly. After an incubation period, which was in two cases 28 days and in the other somewhere between 21 and 28 days, an attack of malaise, anorexia, and fever supervenes, with a lymphangitis of the tissues about the original bite. At the same time erythematous indurated patches may occur irregularly distributed over the skin, and diffuse tender swellings may coexist in the muscles. Such an attack may last a day or two, or even up to a week, but the most interesting part is that a series of them recur at regular intervals, sometimes for several months. The intervals may be as short as three, or as long as ten days. Blood culture and blood film examinations are negative, though a leucocytosis is constantly found when the fever is present, disappearing during the remissions. In one case an erythematous patch was excised, but nothing could be cultivated from it, nor were any organisms found by cutting and staining sections of it. Spontaneous recovery eventually ensues, and no treatment seems to have been found efficacious. Dr. Horder suggests the probability of the infective agent being a protozoon, but says he can bring no evidence in support of this opinion.

---

THE Cerebro-spinal Fluid has been examined a thousand times in two hundred and thirty cases of Acute Septic and Tuberculous Meningitis by Dr. A. Connal, who says that a correct interpretation of the changes which can thus be observed is of great value in diagnosis and prognosis. The mere act of withdrawal is very beneficial in cerebro-spinal fever; but in tuberculous meningitis temporary relief of headache and restlessness is alone secured. In all forms of meningitis the normal transparency of the fluid is lost, and the change may vary from slight turbidity to actual purulence. Increase of turbidity indicates extension of disease. In tuberculous meningitis turbidity is least, and many samples are, in fact, perfectly clear. An appreciable quantity of albumin is always present in the fluid from cases of meningitis; and the proportion should be estimated, because it gives a correct gauge of the acuteness of the meningeal inflammation: here again tuberculosis is exceptional and irregular. The absence of reducing sugar is pathognomonic of meningitis, though it does not follow that if sugar is present meningitis can be excluded. The reappearance of sugar is a sign that the disease is in process of cure. A polymorphonuclear leucocytosis is a sign of cerebro-spinal fever, a mononuclear leucocytosis of tuberculous meningitis. In simple acute cases of the former disease the meningococcus is at first extracellular: it is only later on that the leucocytes ingest it. The presence of large numbers of this organism is an unfavourable sign, and their persistence extracellularly is usually followed by death: this may happen, however, also after they have become intracellular. The tubercle bacillus can be demonstrated in the great majority of cases of tuberculous meningitis, and in increasing numbers as the disease progresses.

---

THE Experimental Production of a Placenta in Animals has been successfully accomplished by Loeb, who recounts some further experiments into the subject in the *Journal of the American Medical Association*. In the guinea-pig any desired number of placentas or deciduomata can be produced by making incisions into the uterus at a certain period after ovulation, and the same holds good of rabbits. The presence of a fertilised ovum is therefore clearly not the cause of the conversion of normal endometrium into decidual tissue. But further research shows that after removal of the ovaries it is very difficult—though not impossible—to produce a deciduoma artificially. It is therefore concluded that the ovaries manufacture some sensitising material, and this is probably produced in the corpus luteum. Experiment also shows that the formation of deciduomata is quite independent of the central nervous system, as it occurs in uteri which have been transplanted into the subcutaneous tissue of the same animal. No explanation is forthcoming of the fact that rupture of a ripe follicle is followed by the formation of a corpus luteum: artificial rupture of a follicle is not able to cause this effect. Whether these researches may prove to have any bearing on the curetting of patients for sterility can hardly yet be predicted; but it seems quite possible that further work on these lines might have very practical applications.

---

SOME important papers on Hydatid Disease are published in the *New Zealand Medical Journal*. That by Professor Barnett, of Otago University, is perhaps the most noteworthy, for it enters fully into the prophylaxis of the disease. First of all he remarks that, though hydatids are prevalent among stock animals in Australia, New Zealand, and the Argentine, it must not be supposed that the public who consume meat from these countries run any risk of the disease; the fear that such an impression might gain ground has, he says, accounted for some reticence as to the exact prevalence of hydatids in those countries. The need for concerted prophylactic regulations is great and is increasing; but if they are adopted it should not be difficult to stamp out the trouble. In Germany such regulations have been enforced for some time, and the result is seen in the fact that the district which has not adopted them harbours six times as many cases per cent. among stock animals as does the rest of the country. In New Zealand hydatids are increasing among human beings: the hospital statistics alone show 83 cases for 1907, treble the number treated in 1891. As notification has only just been made compulsory, it is impossible to estimate exactly how many cases occur in the population as a whole.

---

The chief Problem of Prophylaxis is to prevent the consumption of raw flesh by dogs. It is calculated that about 50 per cent. of stock animals in New Zealand contain hydatid cysts, though, fortunately, their presence in any one organ does not render the whole carcass unfit for food. But the dog alone is the intermediate host, and therefore if dogs can be denied access to the cysts the life cycle of the parasite is necessarily interrupted. The following rules are suggested, to be given to everyone paying a dog license, and to be exhibited in placard form at agricultural and pastoral shows: No dogs to be allowed to feed on raw offal. Unregistered and useless dogs to be killed. Vermifuges to be distributed by veterinary and stock inspectors, and all fæces to be burnt for two days after administration; this involves keeping the dog chained up, of course. All reservoirs to be fenced against dogs. Dogs never to be allowed to lick hands or faces. Uncooked vegetables to be regarded with suspicion. All drinking water to be either boiled or filtered. These regulations are drastic, but in view of the high proportion of hydatid disease among the population it cannot be said that they are unnecessary. A curious case which illustrates the latency of hydatid disease is one reported by Dr. S. C. Godfray, who had a patient brought to him for rupture of a hydatid cyst of the liver during a football match. The case proved fatal, and at autopsy it was shown that a huge cyst had existed near the upper surface of the liver, which had compressed the lung and pushed the heart over to the left; yet notwithstanding this the patient had never sought medical advice nor even complained to his friends, and it was in the act of tackling a fifteen-stone opponent that he sustained the fatal rupture. Dr. Wilson reports six interesting cases of hydatid disease of the lung, all but one of which occurred between the ages of nine and seventeen.

---

The Gastric Crises of Tabetics are always a source of difficulty in treatment, and too often end in the adoption of the morphia habit. Foerster and Küttner, in the *Beit. zur Klinisch. Chirurgie*, advocate for obstinate cases resection of the seventh, eighth, ninth, and tenth posterior dorsal nerve roots. This necessitates laminectomy, an operation of so severe a nature as to prohibit its performance for any but the most serious cases. The patient for whom they were induced to try it was a tabetic of six years' standing, affected with the most persistent and serious attacks of gastric pain and vomiting. For two years he had on an average but six days a month free from a crisis: he was worn to a shadow, and was taking twelve grains of morphia daily. The symptoms were so serious that life was in grave danger, and the resection of several centimetres of each root was performed. Pain and nausea never reappeared after the operation, and his former aversion to food was at once replaced by appetite. The quantity of morphia taken was greatly reduced; four months after operation no gastric symptoms had recurred. A band of anæsthesia encircles the body from three fingers' breadth above the ensiform cartilage to one finger breadth above the umbilicus. The other symptoms of tabes remain as before.

---

Captain Hamilton, of the Indian Medical Service, has seen a good deal of Infantile Enteric Fever, and he sums up his observations in a contribution to the *Indian Medical Gazette*. Enteric before the age of two years is by no means so uncommon as most textbooks allege: many cases are mild (amongst native Indians at least) and pass undiagnosed. The prognosis is correspondingly good, if the disease is recognised and properly treated. The best diet is whey: good nursing, plenty of water, and as few drugs as possible are also important. The very closest watchfulness is essential; twice a day is the minimum, and three visits are preferable. Complications are much less common than in adults; heart failure from prolonged pyrexia is most to be apprehended, and good brandy not too dilute is valuable for this. The author fancies that European children are less addicted to the very mild forms of ambulatory typhoid which he has seen in Asiatics, some of whom are able to run about and to play even at the height of the illness. Finally, he notes that infants during this disease frequently increase in stature in the most extraordinary way.

---

Some novel suggestions for the Treatment of Delirium Tremens are those of Dr. G. E. Pettey, who ascribes the symptoms of this condition to the accumulation of toxic products, autogenous as well as alcoholic, in the blood. Accordingly he aims at the removal of these deleterious substances. He gives normal salt solution in large quantities by the rectum, hypodermically, or, if necessary, intravenously. Thus the entire circulatory system is flushed with fluid to its utmost capacity, and this is then relieved by free purgation with large and repeated doses of Epsom salts. Calomel in full doses is also given. Sparteine is administered in two-grain doses for the purpose of supporting the heart and promoting diuresis. For the delirium itself gelsemine is given every hour, or every two hours, until its physiological effect is produced: the dose advised is $\frac{1}{25}$ grain. Alcohol is reduced to moderate limits, but is not entirely withdrawn: opium and other narcotics are condemned, as not merely dangerous but useless. Physical restraint is also held to be not permissible. In 450 consecutive cases the results of this line of treatment are described as excellent, and no death from delirium tremens occurred in the whole series.

